# Harnessing Neuroendocrine and Circadian Clocks in Beneficial Insects for Agricultural Chrono-Management

**DOI:** 10.1021/acs.jafc.6c12791

**Published:** 2026-10-20

**Authors:** Xianguo Zhao, Yudan Jian, Su Wang

**Affiliations:** Institute of Plant Protection, 107624 Beijing Academy of Agriculture and Forestry Sciences, Beijing 100097, China

**Keywords:** beneficial insects, neuroendocrine regulation, circadian clocks, agricultural chrono-management

## Abstract

Pollinators and natural enemies provide essential agricultural ecosystem services that are regulated by circadian clocks, hormones, and biogenic amines. This review synthesizes research on the integration between neuroendocrine systems and circadian clocks across different taxa, identifying both conserved regulatory mechanisms and lineage-specific adaptive traits. These multilayered interactions coordinate insect behavior, life-history development, and the alignment of insect activities with available resources. We propose agricultural chrono-management, a framework to improve ecosystem services by using targeted environmental cues to align beneficial insect activity with farming schedules. However, most evidence so far comes from the laboratory and controlled studies, and field validation remains limited. We further evaluated the feasibility, ecological risks, and climate resilience of photoperiodic regulation, nutritional modulation, and synthetic ecological interventions. Key future research priorities include multiomics integration, large-scale field validation, and predictive modeling of ecosystem services. This synthesis links molecular-level regulatory mechanisms to ecological outcomes and establishes a foundational framework for climate-resilient management of beneficial insects.

## Graphical abstract

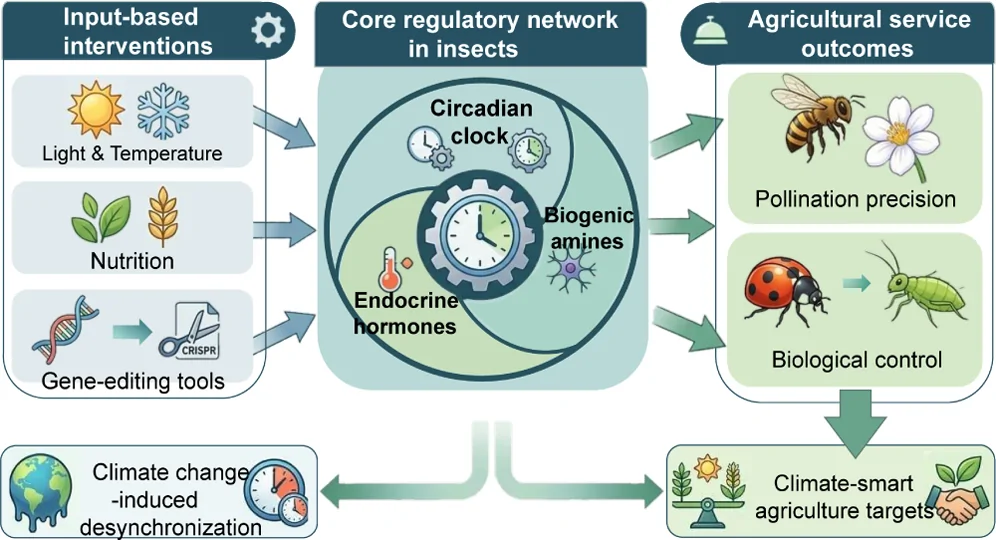

## 1 Introduction

Beneficial insects are keystone components of terrestrial ecosystems, providing pollination and biological pest control services that sustain agricultural productivity and ecological resilience (Figure 1). Pollinators such as honey bees, bumblebees, and hoverflies directly improve crop yield and quality, while natural enemies including lady beetles and parasitoid wasps suppress herbivorous pests and reduce reliance on chemical pesticides. Global assessments, including the IPBES report, estimate that the annual economic value of these services amounts to hundreds of billions of US dollars. Despite their enormous significance, beneficial insects face growing threats from multiple interacting stressors, most notably climate change, habitat fragmentation, and pesticide exposure. All of these factors undermine both population stability and the reliable provision of ecosystem services.^1–3^

**Figure 1:**
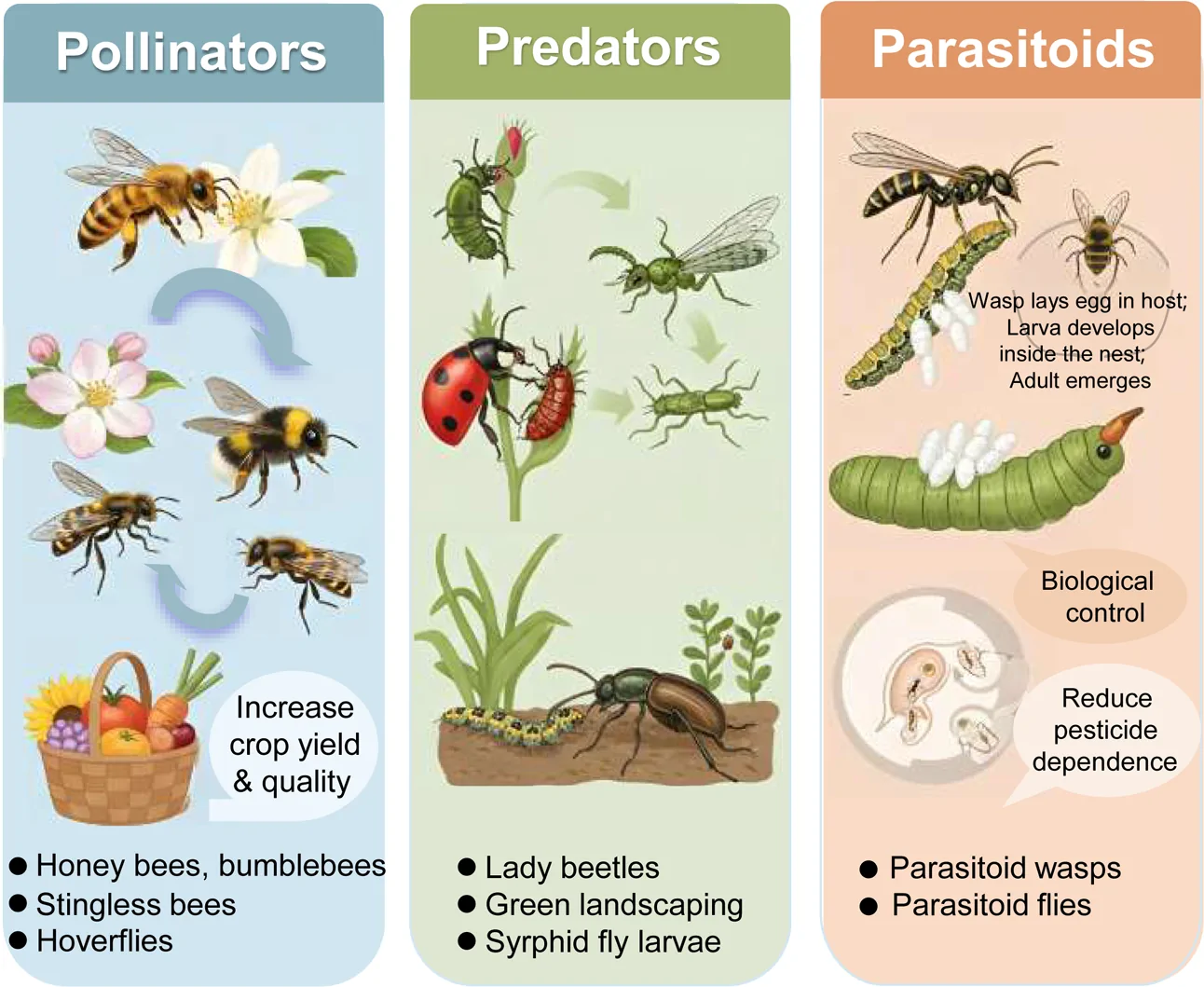
Schematic illustration of the key roles of beneficial insects in sustainable agriculture. The left panel shows the pollinators boosting crop yield and quality. The middle panel presents the predatory insects achieving natural pest control via optimized ecological landscaping. The right panel demonstrates the parasitoids delivering effective biological pest control, which in turn reduces reliance on pesticides.

Forecasting insect population responses to global change and designing effective management strategies require a clear understanding of how insects adjust their phenology and behavior to changing environmental conditions. Climate drivers of adult insect activity are shaped by a species’ life history traits, which provides a foundation for predicting species-level responses to environmental change.^4^ For parasitoid wasps, which are key natural enemies enabling biological pest control, identifying their adaptive strategies can further inform the development of improved management approaches.^5^ Their adaptive responses are governed by three intrinsically linked regulatory systems: biogenic amines, endocrine hormones, and the circadian clock. Studies on model insects, particularly bees, have shown that environmental stressors profoundly alter foraging behavior and traits at the colony level.^6^ At the physiological level, insects display sophisticated network integration: the stress response and immune system share and reconfigure molecular components to optimize organismal defense.^7^ Even so, the functional integration of these systems in beneficial insects under ecologically realistic conditions with multiple co-occurring stressors remains poorly understood. Mechanistic insights obtained from simplified laboratory models often fail to account for ecological complexity and lineage-specific adaptations, limiting their extrapolation to nonmodel beneficial insect species. This gap hinders a holistic understanding of insect resilience and obstructs the development of management strategies informed by physiological mechanisms.

Biogenic amines, particularly octopamine and tyramine, act as neuromodulators that regulate the behavioral state and physiological switching in invertebrates.^8^ In honey bees, octopamine enhances foraging sensitivity, aggression, and memory formation,^9–12^ while at the parasitoid–host interface, differences in the host-search performance of parasitoid wasps trigger octopamine-mediated suppression of oviposition in host Drosophila.^13^ Endocrine hormones, particularly juvenile hormone (JH), ecdysteroids, and neuropeptides, act over longer time scales to control developmental transitions, reproduction, and behavioral specialization.^14–16^ In eusocial pollinators, JH governs worker task differentiation, with elevated hormone levels promoting the transition to foraging roles in honey bee workers.^17,18^ In contrast, seasonal hormonal shifts in many predatory beetles, particularly JH signaling, mediate transitions between resident predation and migratory dispersal.^19^ Neuropeptides such as allatostatins and allatotropins further integrate nutritional and environmental signals, enabling precise regulation of physiological states in sync with resource availability and environmental periodicity (Table 1).

**Table 1:** Advances in Insect Physiology Research: Historical Focus and Relevance to Beneficial Insect**s**

System	Primary historical focus	Key methodological innovations	Relevance to beneficial insects
Biogenic amines	Regulation of aggression, learning, and motivational control^10,36,37^	HPLC, electrophysiological recording, and neuropharmacological assays	Enhancement of foraging efficiency, modulation of aggression, and regulation of predatory motivation^10,13,36^
Endocrine hormones	Control of development, reproductio,n and diapause^14,38^	Hormone titration assays and hormonal analogue feeding	Regulation of task allocation, developmental transitions, and reproductive maturation^14,39,40^
Circadian clocks	Coordination of diel and physiological rhythms across seasons^40,41^	Gene expression profiling, locomotor activity tracking, and chronobiological modeling	Temporal alignment with floral phenology and availability of prey^40–43^

Circadian clocks, driven by transcriptional–translational feedback loops involving conserved genes such as period (*per*), timeless (*tim*), clock (*clk*), cycle (*cyc*), and cryptochrome (*cry*), regulate daily behavioral and physiological rhythms.^20^ These clocks confer significant fitness advantages when their period resonates with environmental cycles, underscoring their broad ecological relevance across diverse taxa.^21^ In pollinators, circadian control aligns foraging activity with the availability of floral resources and also supports foraging orientation and navigation.^22^ In natural enemies, circadian timing synchronizes predatory or parasitoid activity with the availability of hosts or prey, ultimately enhancing the efficiency of biological control.^23^ The circadian clock has been recognized as a powerful tool for behavioral manipulation in biocontrol management as synchronous temporal organization between natural enemies and their targets is essential for successful predator–prey associations.^23,24^ Disruption of circadian regulation caused by artificial light, altered photoperiod cues, or agrochemical exposure reduces the functional performance and ecological effectiveness of these beneficial insects.^23,25–27^

Although each of these regulatory systems has been studied individually, the mechanisms linking hormonal modulation of circadian outputs, aminergic control of hormonal signaling, and clock regulation of neurochemical rhythms remain incompletely understood in beneficial insects. A growing body of evidence indicates the existence of multilayered bidirectional interactions among these systems: JH alters temporal activity profiles in honey bees, circadian transcription factors influence octopamine biosynthesis, and amine–hormone cross-talk modulates stress responses in both pollinators and predators.^28–33^ These findings suggest that the behavioral plasticity of beneficial insects in dynamic agricultural environments is best understood through an integrated neuroendocrine–circadian framework: the circadian clock integrates photoperiodic and thermal cues,^34^ while JH and ecdysteroid pathways interact bidirectionally with the circadian system.^35^ In pollinators, this integrated regulation enables time-memory for floral resources and supports time-compensated sun-compass navigation.^22^

Research on biogenic amines, hormones, and circadian clocks progressed along separate disciplinary trajectories for most of the twentieth century before converging with the rise of molecular genetics and high-throughput omics. Early endocrinological studies identified JH and ecdysteroids as core regulators of insect development,^44^ while advances in neurochemistry clarified the molecular architecture of biogenic amine signaling pathways, including the receptors for octopamine, dopamine, and serotonin.^31^ The discovery of core clock genes in *Drosophila* during the 1980s and 1990s, which culminated in the characterization of the *per* gene^45^ and the *tim* gene,^46^ laid the molecular foundation for the field of chronobiology. Over the past two decades, integrative research covering all three systems in beneficial insects has accelerated, as summarized in Table 1.

Despite notable progress, substantial knowledge gaps remain. Comparative studies across different species are scarce, making it difficult to determine whether mechanistic insights obtained from model organisms can be extrapolated to beneficial insects occupying diverse ecological niches. The core conserved system governing insect neuroendocrine timing comprises the circadian clock and its light input.^47^ However, laboratory experiments cannot replicate the environmental heterogeneity present in natural habitats, especially the fluctuating thermal, photoperiodic, and nutritional conditions that insects experience in the wild, leaving unclear how neuroendocrine and circadian interactions function under ecologically realistic settings. Field-level manipulations targeting the neuroendocrine and circadian systems of beneficial insects have remained scarce. Although laboratory studies have documented the effects of JH analogues such as methoprene on beneficial insects,^48^ their broader ecological impacts under field conditions remain largely unknown. Similarly, molecular-level research on nontarget beneficial insects, especially non-Apis bees, remains limited,^49^ leaving a critical gap in our understanding of how these interventions function under ecologically realistic conditions. Furthermore, the integration between molecular-scale research and applied agricultural practice is limited, which hinders the translation of mechanistic insights into ecologically sound pest- and pollinator-management frameworks. A further complication is provenance: part of the causal evidence for these interactions comes from laboratory models and pest taxa rather than from beneficial insects, a problem that is audited explicitly in Section 3.

This review addresses these gaps by synthesizing the current understanding of integrated aminergic, endocrine, and circadian regulation in beneficial insects. It traces the conceptual development of this research area, evaluates existing mechanistic models, and compares regulatory architectures across pollinators and natural insect enemies. A core objective of this work is to establish the conceptual and practical foundations for agricultural chrono-management (ACM). We define ACM as a conceptual and applied strategy that deliberately manipulates environmental cues, specifically the photoperiod, temperature, and nutrition, to modulate the internal neuroendocrine and circadian systems of beneficial insects. This allows precise temporal alignment of beneficial insect activity with crop phenology and pest susceptibility windows across both diel and seasonal time scales.

ACM does not replace existing management frameworks but rather adds a complementary temporal dimension. Integrated pest management (IPM) determines which control tactics to deploy based on pest damage thresholds, while precision agriculture optimizes the location and intensity of agricultural inputs through spatial sensing and variable-rate technologies. ACM introduces a proactive temporal focus: it addresses not only what interventions to implement and where to implement them but also when beneficial insects are physiologically primed for peak ecosystem service delivery and when pests are at their most vulnerable. Building on this foundation, ACM proposes manipulating environmental cues so as to entrain these biological rhythms to crop phenological schedules. At present, the framework remains largely hypothesis-driven: field-level manipulations are scarce, and both open-field scalability and ecological consequences await rigorous validation. By shifting the focus from passive conservation to active coordination of insect-mediated ecosystem services, ACM integrates a timing dimension that enhances both the IPM and precision agriculture. This temporal alignment has the potential to improve the efficiency, reliability, and resilience of insect-mediated services in the agricultural landscape.

Most critically, the circadian regulation of pest physiology and behavior is an equally essential, yet often overlooked, component of ACM. Core pest activities including host-seeking, feeding, mating, and egg hatching are all gated by endogenous circadian clocks. As a result, mismatches between pest activity rhythms and management interventions are a primary driver of IPM inefficiency. For example, the circadian clock regulates pesticide responses via the conserved Pdp1 pathway,^50^ providing a mechanistic foundation for the hypothesis that circadian-mediated modulation of detoxification gene expression can substantially increase the pesticide dose required to achieve equivalent pest lethality when pesticides are applied outside the pest’s vulnerability window. Meanwhile, pesticide applications made at midday, which coincide with peak pollinator activity, substantially raise the risk of nontarget exposure.^51^

Crucially, these diel rhythms do not operate as independent processes but are hierarchically nested within broader seasonal and climatic timing systems. Seasonal accumulation of photoperiod cues (e.g., shortening day length in autumn), cumulative degree days, and interannual climate variability act as upstream signals that calibrate diel clock outputs to regulate fitness-critical life transitions: diapause initiation and termination, migration, reproductive dormancy, and colony founding in eusocial species. For beneficial insects, these transitions are closely tied to crop phenology and pest population dynamics; for instance, diapause termination in predatory beetles is timed to occur before spring aphid outbreaks, whereas swarming in honey bees is synchronized with regional peaks in floral resource availability. Effective chrono-management therefore requires simultaneous optimization of temporal alignment across three trophic levels (pests, beneficial insects, and crops) at both diel and seasonal scales to maximize pest suppression while minimizing collateral impacts on surrounding ecosystems (Table 2).

**Table 2:** Conceptual Boundaries: ACM versus IPM and Precision Agriculture

Dimension	IPM	Precision Agriculture	ACM
Primary question	Which tactics to employ?	Where and how much to apply?	When are organisms physiologically primed?
Decision basis	Pest counts, economic thresholds	Sensor data, GPS, remote sensing	Circadian/seasonal rhythms of pests, beneficials, crops
Approach to beneficial insects	Passive conservation; minimize harm	Spatial targeting to reduce drift	Proposed active orchestration via the clock/hormone entrainment, pending field validation
Temporal scope	Reactive; threshold- or calendar-triggered	Real-time but not biologically timed	Proactive; calibrates diel–seasonal rhythms of three trophic levels
Mechanistic foundation	Ecological observation, population models	Engineering, data science	Neuroendocrine–circadian biology
Relationship to ACM	Provides a tactical toolkit that ACM temporally optimizes	Provides the sensing/delivery infrastructure that ACM can leverage	Adds the timing dimension to both

Beyond agricultural applications, the synthesis presented in this paper establishes a scalable paradigm for understanding how environmental information is transduced into coordinated behavioral responses across diverse taxa. The integrated framework connecting molecular clocks, hormones, and neurotransmitters mirrors regulatory architectures observed in vertebrate systems, including the hypothalamic–pituitary–adrenal control of stress responses and the photoperiodic regulation of reproduction. Beneficial insects therefore provide tractable experimental models for investigating the general principles of behavioral plasticity under environmental change with broader relevance to the fields of comparative physiology and conservation biology.

Even with the notable advances achieved in research on model insect species, three critical knowledge gaps remain: the interactions between circadian clocks and hormones in nonmodel beneficial insects remain underexplored, field-level validation of these integrated mechanisms is scarce, and translational applications of related findings to agricultural practice remain at an early stage. This review addresses these gaps by establishing a hierarchical model that spans scales from the molecular level to the ecosystem level.

## 2 Historical and Theoretical Foundations

To frame the mechanistic analysis that follows, this section traces how research on neuroendocrine and circadian regulation has evolved from examining each system in isolation to viewing them as components of an integrated network.

### 2.1 Historical Development and Functional Evolution of Biogenic Amines

To contextualize these integrative mechanisms, we first outline the historical and theoretical development of research on biogenic amines. Investigations on biogenic amines in insects started in the 1950s and 1960s, after octopamine, dopamine, and serotonin were discovered in invertebrate nervous systems. Octopamine was first identified in octopuses and then detected in insects and acts as the invertebrate counterpart of vertebrate noradrenaline (norepinephrine). It modulates locomotor activity by acting on neuromuscular junctions, where it potentiates excitatory junction potentials via presynaptic octopamine receptors.^52^

Behavioral assays in bumblebees have demonstrated that reward-induced positive affective states can be transferred between conspecifics, modulating judgment bias and foraging-related decision making.^53^ Dopamine was first found to regulate insect salivary gland secretion through cAMP-dependent pathways.^54,55^ Subsequent studies demonstrated that it also mediates aversive learning across multiple insect species^56–58^ and is involved in behavioral switching^59^ and phase-related dispersal plasticity.^60^ Serotonin, first linked to gut physiology in insects,^61^ also modulates neural energetic states in aggression-relevant brain regions of honey bees.^62^ Although these monoamines share common biochemical pathways, they have evolved distinct adaptive specializations: octopamine modulates reward representation during honey bee dance communication;^63^ dopamine delivers instructive signals for both appetitive and aversive learning, which overturned the earlier model that octopamine mediates reward and dopamine mediates punishment;^64,65^ and serotonin regulates circadian entrainment to light through the d5-HT1B receptor in clock neurons.^66^

### 2.2 Classical Models of Insect Hormones

The discovery of JH and ecdysteroids established that endocrine regulation is central to insect development and life cycle transitions.^16,67,68^ JH not only orchestrates developmental transitions including metamorphosis but also mediates behavioral transitions, such as the nurse-to-forager shift in honey bees.^69–71^ Ecdysteroids, particularly 20-hydroxyecdysone (20E), are key endocrine drivers of diapause termination,^38^ enabling insects to synchronize life cycle transitions with seasonal resource availability.

Allatostatin C suppresses JH biosynthesis in the corpora allata, thereby fine-tuning JH titers to coordinate life-history transitions. In *Dendroctonus armandi*, knockdown of DaAST elevates JHAMT expression, increases pupal mortality, and reduces emergence rates.^72^ Parasitoid wasps deliver a venom composed of proteins and peptides during oviposition to regulate the host physiology. In *T. sinensis*, multiomics analyses have identified 143 venom proteins and 52 putative transcripts, most of which are hydrolases and protease inhibitors.^73^ Taken together, these hormonal systems orchestrate life-history transitions and behavioral trade-offs in alignment with resource dynamics, a phenomenon termed life cycle coupling.

### 2.3 Circadian Clock Genes and Rhythmic Adaptation

Molecular studies in *Drosophila* have established a conserved core clock composed of *per*, *tim*, *clk*, and *cyc*, with the light-sensitive *cry* serving as a blue-light photoreceptor that triggers TIM degradation to reset the clock.^74^ These genes form a transcriptional–translational feedback oscillator that maintains synchrony with environmental cycles, particularly light/dark cycles.^75^ In *Apis mellifera*, time-restricted foraging induces significant phase shifts in the cycling of core clock genes *per* and *cry2* across brain, subesophageal ganglion, and antennal tissues, demonstrating that foraging time itself functions as a zeitgeber.^76^ In *Bombus* spp., foragers maintain robust diurnal rhythms even under Arctic permanent daylight, with circadian clocks entrained by daily cycles in light intensity and temperature.^77^ In natural enemies such as *Trichogramma*, photoperiodic regimes modulate the expression of core clock genes, thereby regulating diapause and fecundity.^78^ This provides a mechanistic basis for the circadian control of adult emergence rhythms as *Trichogramma* adults emerge in the early morning hours after light onset.^79^ The circadian oscillator modulates secretion of JH and ecdysteroid in insects.^35^ In *Pyrrhocoris apterus*, photoperiodic responses of the chronome correlate with developmental pathway switches.^80^

### 2.4 Early Theoretical Models of System Interaction

Although the three systems have been historically studied in isolation, they are now recognized as functionally interconnected. Circulating levels of many insect hormones, including JHs and ecdysteroids, are modulated by the circadian system.^35^ In honey bees, octopamine and tyramine modulate sucrose responsiveness, affecting behavioral development and division of labor.^10^ One foundational model provides complementary interpretations of the interactions. The feedback network model proposed an integrated system with bidirectional interactions: biogenic amines alter clock gene expression, while hormones concurrently adjust the sensitivity of amine receptors.^10^ Although these early models captured bidirectional interactions, they lacked ecological context. Contemporary frameworks now conceptualize these systems as a tightly interconnected regulatory network that responds dynamically to photoperiodic cues and resource dynamics, as illustrated in Table 3.

**Table 3:** Representative Models of Single and Interactive Regulatory Systems in Beneficial Insect**s**

Category	Model name	Organism	Key factors	Main conclusions	Limitations	Direct references
Biogenic amines	Octopamine reward	*A. mellifera*	Octopamine and sucrose responsiveness	Mdulation of foraging motivation and associative learning	Context-dependent outcomes	^81–83^
Biogenic amines	Dopamine reinforcement	*Bombus terrestris*	Dopamine and reward valuation	Drives the dopamine-dependent positive emotion-like state	Validated primarily under laboratory conditions	^84^
Hormones	JH task allocation	*A. mellifera*	JH titer and worker age	Initiates transition to foraging	Influence of colony status	^70,85^
Hormones	JH-mediated diapause	*Harmonia axyridis*	JH; photoperiodic accumulation; prey availability	Diapause termination is gated by photothermal and trophic cues	High sensitivity to unseasonal warming	^86,87^
Circadian clocks	*per* and *cry* feedback loop	*A. mellifera* and *Bombus* spp	*per*, *cry*, and *clk*	Governs daily rhythmicity and temporal behavioral outputs	Exhibits interspecific variability	^35,88^
Interaction	Feedback network	Bees	Octopamine, JH, and *per*	Cross-talk between biogenic amines and circadian genes	Mechanistic pathways remain unclear	^35,82^

## 3 Mechanistic Interaction Networks

Building on the historical foundations outlined in Section 2, this section examines the bidirectional regulatory networks connecting circadian, hormonal, and aminergic systems. The evidence assembled in this section is of two types. Category 1 is direct evidence from beneficial insects, namely, pollinators (*A. mellifera*, *Bombus terrestris*) and natural enemies (*Harmonia axyridis*, *Trichogramma* spp.), and is treated as transferable to ACM. Category 2 refers to mechanistic evidence obtained from taxa that provide neither of these two ecosystem services (*Drosophila melanogaster*, *Periplaneta americana*, *Blattella germanica*, *Bombyx mori*, *Antheraea pernyi*, *Manduca sexta*, and *Agrotis ipsilon*). This evidence serves only one purpose: to identify which nodes and causal links are contained within the bidirectional regulatory network through the loss of function and rescue experiments. It is never used to infer the behavior or field performance of the beneficial insects. The provenance, ecological status, and validation status of each taxon are reviewed in Sections 3.4.

### 3.1 Circadian–Hormonal Bidirectional Regulation: a Two-Coupled-Loop Feedback Framework

This bidirectional architecture is schematically illustrated in Figure 2. Circadian and endocrine systems interact with each other: circadian timing modulates hormone biosynthesis and the expression of hormone-related genes, indicating that rhythmic physiological processes and behaviors are not regulated by independent systems.^35^

**Figure 2:**
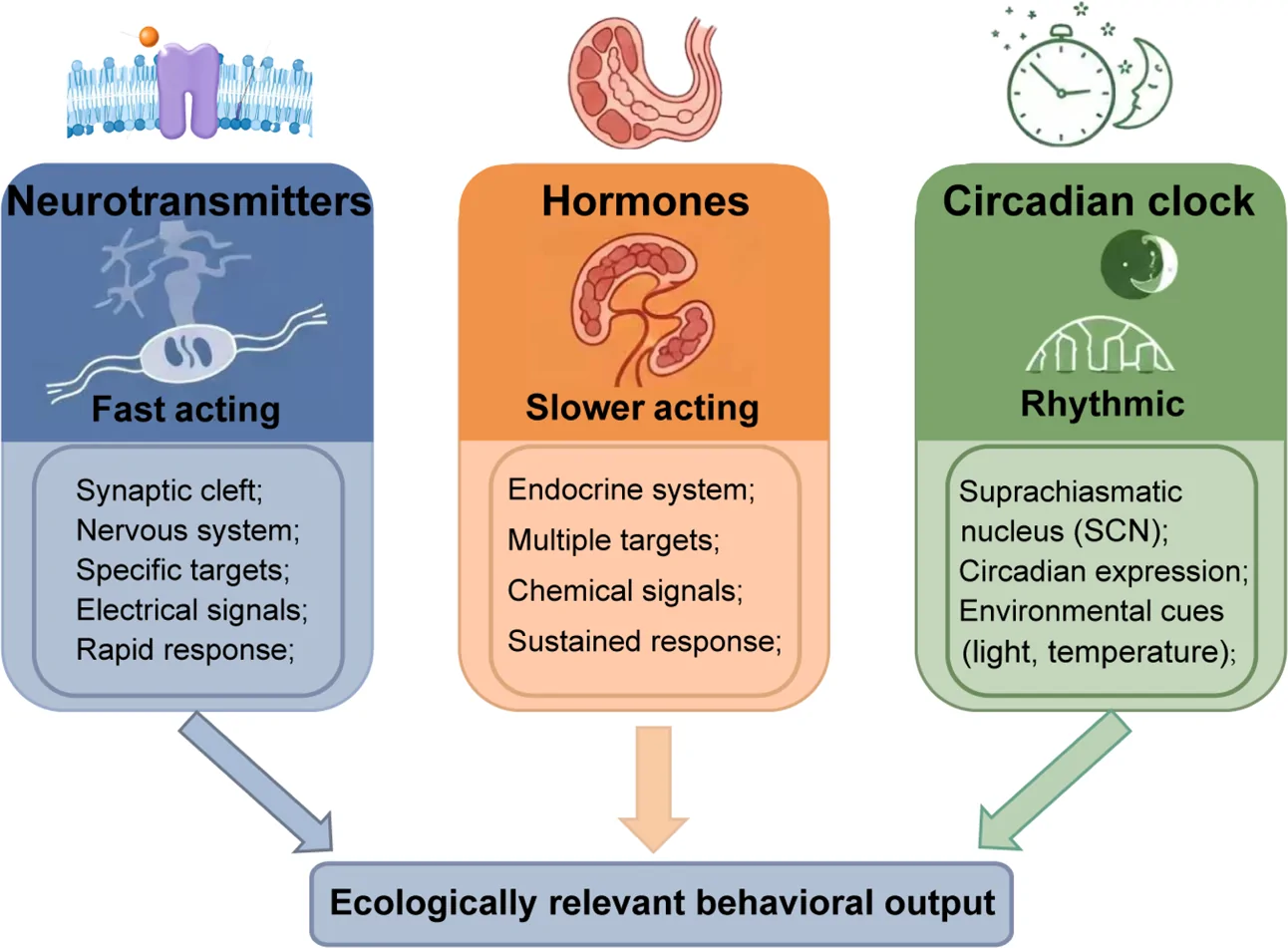
A conceptual model of multiscale physiological regulation in insect ecology. The schematic contrasts three core regulatory systems: fast-acting neurotransmitters that drive immediate neural responses via synaptic transmission, slower-acting hormones that coordinate systemic physiological changes through the endocrine system, and the circadian clock that imposes rhythmic constraints based on environmental light cues. These integrated pathways collectively dictate the final ecological behavior output, highlighting the hierarchical transition from molecular signaling to organismal performance.

The positive regulators of the core circadian clock, CLK and CYC, act outside their canonical timekeeping role to drive JH biosynthesis in the corpora allantae. Knockdown of *clk* or *cyc* blocks short-day-induced reproductive diapause in the ladybird *H. axyridis*. CLK and CYC physically interact with the NuA4/TIP60 chromatin acetyltransferase complex to stimulate local JH production in the corpora allata, and methoprene, a JH agonist, rescues the diapause phenotype caused by *clk/cyc* knockdown.^89^ This finding reveals a noncanonical, photoperiod-sensitive transcriptional module that connects circadian proteins to endocrine output.

Conversely, JH feeds back to reshape the strength and ontogeny of circadian rhythms (loop B). In *B. terrestris*, experimental reduction of JH weakens the development of circadian rhythms in newly emerged workers, whereas JH-III replacement therapy restores rhythm strength, indicating that JH exerts an organizational effect on the maturation of the circadian system.^90^ JH manipulation increases the strength of circadian rhythms and accelerates rhythm development but does not alter the free-running period, overall locomotor activity, or sleep architecture,^90^ suggesting that JH modulates the physiological output of the clock rather than the core pacemaker itself.

Together, Loops A and B constitute a bidirectional, multilayer regulatory network: the core clock machinery sets JH titers via a noncanonical transcriptional route, while JH in turn tunes the amplitude and developmental trajectory of circadian rhythms. Biogenic amines act upstream of Loop B, modulating *per* gene expression and locomotor output downstream of the core oscillator.^28,82^ This framework moves beyond phenomenological correlation to establish a mechanistically anchored model of neuroendocrine–circadian integration.

This bidirectional architecture has been empirically observed in beneficial insects with disparate social and ecological traits. In honey bees, JH titers show significant diurnal variation,^91^ and accumulating evidence indicates that JH biosynthesis is under circadian control, with farnesol oxidase identified as a key regulator of this process.^35^ This endocrine oscillation is intertwined with behavioral rhythms as manipulation of light–dark cycles can reverse or reshape honey bee activity patterns.^92^ Extending from diel to seasonal time scales, the photoperiod–JH axis regulates diapause induction: short autumn daylengths suppress JH biosynthesis via transcriptional regulation, triggering metabolic reprogramming for overwintering.^86,93,94^ In *H. axyridis*, circadian clock genes *clk*/*cyc* modulate JH production via the NuA4/TIP60 complex to determine diapause entry.^89^ 20-Hydroxyecdysone (20E)-mediated diapause termination is gated by the photoperiodic and circadian system, which acts as an endocrine switch in which ecdysteroid biosynthesis is under circadian control,^95^ and by cumulative temperature thresholds. In Trichogramma, spring warming accelerates diapause termination and enhances parasitism efficiency, thereby aligning wasp emergence with host egg availability.^96,97^ However, climate warming causes phenological mismatches between natural enemies and their prey or hosts, disrupting life cycle synchronization and reducing biocontrol efficacy;^98^ at the population level, warming also enables pest range expansion, allowing pests to escape their coevolved natural enemies.^99^

Taken together, these findings demonstrate that the two-loop feedback framework operates consistently across both diel and seasonal scales: at the diel level, the clock modulates JH biosynthesis (loop A) and JH in turn tunes the amplitude and ontogeny of circadian rhythms, with biogenic amines modulating *per* expression downstream; at the seasonal level, photoperiod-regulated *clk*/*cyc* expression sets JH titers via NuA4/TIP60 to gate diapause entry (loop A instantiated at a life-history transition), while circadian-gated ecdysteroid output coordinates diapause termination with temperature accumulation.^89,95^ This multiscale coherence is precisely what allows augmentative biological control to manipulate environmental cues and align beneficial insect activity with crop phenology and windows of pest susceptibility.

### 3.2 Circadian–Aminergic Bidirectional Regulation

The circadian clock regulates the synthesis and turnover of major biogenic amines, controlling the aminergic tone at both the transcriptional and enzymatic levels. In *A. pernyi* (a sericultural lepidopteran and therefore outside the pollinator/natural-enemy definition used here), the arylalkylamine *N*-acetyltransferase gene (*nat*) encodes the rate-limiting enzyme for melatonin biosynthesis. RNA interference targeting *clk* or *cyc* suppresses *nat* transcription, whereas RNAi targeting the repressor *per* upregulates this transcription. This indicates that the CLK/CYC heterodimer directly drives *nat* expression as an output of the circadian clock.^100,101^ In *B. mori* (the domesticated silkworm; likewise outside that definition), CLK/CYC drives BmaaNAT expression to regulate temperature-dependent diapause.^102^ This clock-controlled *nat* serves as a critical link between the circadian system and the endocrine switch that releases PTTH to regulate pupal diapause.^100^ In *P. americana* (an urban cockroach pest, used here as a neurophysiological preparation), knockdown of aaNAT1 abolishes locomotor rhythmicity under both light–dark cycles and constant dark conditions, and continuous melatonin administration restores this rhythmicity,^103^ which causally confirms that aaNAT is a clock output gene that feeds back into the circadian system. Regarding dopamine biosynthesis, the mRNA levels of two dopamine biosynthetic enzymes, tyrosine hydroxylase and dopa decarboxylase, exhibit circadian rhythmicity in the heads of male *B. germanica* (also an urban cockroach pest). Exogenous dopamine alters total locomotor activity without shifting the underlying circadian rhythm,^104^ indicating that dopamine acts downstream of the circadian pacemaker.

Biogenic amines act back on the circadian system to modulate clock neuron output. In *Drosophila* (a laboratory model species, not a beneficial insect), the wake-promoting large ventral lateral neurons (l-LNvs), which form a subset of clock neurons, elevate cAMP levels in response to both dopamine and octopamine. Light suppresses both dopamine- and octopamine-induced cAMP responses through distinct mechanisms: suppression of the dopamine response is mediated by light-induced upregulation of inhibitory D2-like receptors, while suppression of the octopamine response is gated by the circadian clock protein PER, which specifically amplifies octopamine-induced responses at night.^105^ This forms a bidirectional gating loop: the clock sets the phase of aminergic sensitivity and amines in turn modulate the output gain of clock neurons. *Ebony*, an *N*-β-alanyl-biogenic-amine synthetase, is rhythmically expressed in glia downstream of the clock and is required for normal circadian locomotor rhythms. Ebony-expressing glia are located adjacent to dopaminergic and serotonergic neurons, and genetic interaction analyses with the dopamine transporter gene confirm that *Ebony* is involved in dopaminergic signaling.^106^ These findings position aminergic metabolism as an essential relay between the molecular oscillator and behavioral output.

Collectively, these findings confirm that the relationship between the circadian clock and biogenic amines is reciprocal:^35^ the clock drives rhythmic amine biosynthesis through effector genes containing E-box elements, while amines in turn feed back to regulate and shape the output of clock neurons. In honey bees, octopamine modulates *per* gene expression and locomotor behavior without altering the core pacemaker,^82^ demonstrating that this bidirectional regulatory architecture is conserved in this ecologically important pollinator. This relationship parallels and interconnects with the clock–hormone regulatory loops introduced in Section 3.1.

### 3.3 Hormone–Amine Bidirectional Metamodulation

Biogenic amines and endocrine hormones participate in a second-order neuromodulation process termed metamodulation, and this interaction itself follows a bidirectional regulatory relationship.^107^ Steroid hormones shape the baseline activity of aminergic signaling. In *M. sexta* (a solanaceous crop pest and classical neuroendocrine model), the preadult surge of 20E is both necessary and sufficient to explain the increased octopamine levels observed in adult individuals. When researchers blocked the ecdysteroid source via ligation and subsequently infused 20E, tyramine β-hydroxylase (TβH) levels were restored. Additionally, 20E promotes somatic growth of median octopaminergic neurons and drives the late-life expression of octopamine-immunoreactivity in newly differentiated lateral neurons.^107,108^ These findings confirm that ecdysteroid signaling establishes the aminergic substrate required for the development of adult behaviors. At the membrane level, the G protein-coupled receptor DopEcR can bind both 20E and dopamine, allowing it to integrate steroid and aminergic signals. In moth *A. ipsilon* (a noctuid crop pest), AipsDopEcR is predominantly expressed in the antennal lobe and mushroom bodies. RNAi-mediated silencing of AipsDopEcR eliminates the behavioral response to sex pheromone in wind tunnel experiments.^109,110^ This result reveals that DopEcR acts as a molecular coincidence detector through which 20E and dopamine synergistically regulate behaviorally relevant neural circuit output.

Aminergic signaling feeds back to regulate the endocrine status. In honey bees, octopamine administration accelerates the nurse-to-forager transition and increases JH titers, and this effect can be blocked by octopamine receptor antagonists. Conversely, JH treatment alone is sufficient to induce foraging behavior.^82^ This forms a positive feedback loop: JH promotes the behavioral transition to foraging, a process accompanied by increased octopamine levels, and octopamine in turn reinforces the JH-driven behavioral program. In *A. mellifera*, JH titers oscillate in phase with *per* mRNA expression in foragers,^91^ which places the amine–hormone feedback loop directly within the clock-output pathway. In honey bee larvae, octopamine and serotonin stimulate JH release from the corpora allata in a dose-dependent manner in vitro, increasing the levels of both JH and its precursor methyl farnesoate.^111^ This confirms that direct aminergic-to-endocrine feedback acts at the level of hormone production.

Bidirectional integration across the three systems: The metamodulatory relationship spans all three regulatory layers. In *A. mellifera*, the octopamine-dependent modulation of per expression and locomotor behavior described in Section 3.2 ^82^ converges with the JH–*per* coupling outlined above.^91^ In *H. axyridis*, the CLK/CYC–NuA4/TIP60 complex regulates JH titers,^89^ which in turn control the developmental transition to diapause. During reproductive care in insects, dopamine and JH are jointly upregulated, indicating that the aminergic and endocrine systems are corecruited by the same developmental switch.^112^ Finally, in *M. sexta*, 20E not only increases octopamine levels but also regulates the somatic growth of octopaminergic neurons,^108^ placing hormonal regulation upstream of the aminergic circuit architecture.

Taken together, Sections 3.1–3.3 form a three-node bidirectional network: clock genes regulate JH biosynthesis and aminergic enzyme expression (via E-box-containing genes such as *nat*); JH and 20E in turn adjust the amplitude and ontogeny of circadian rhythms and establish the baseline activity of aminergic signaling; and biogenic amines modulate *per* expression, regulate clock neuron output, and reinforce hormone-driven behavioral programs. No connection in this network is unidirectional, and it is precisely this multilayered reciprocity that underlies the plasticity of beneficial insect behavior in dynamic agricultural environments. Since several causal links in this network were established in category 2 taxa, this scheme should be interpreted as a mechanistic scaffold whose nodes still require direct verification in beneficial insects, rather than as a characteristic that has already been confirmed in pollinators and natural enemies.

### 3.4 Multilevel Regulatory Cascades Connecting Molecular Mechanisms to Ecological Functions

The interactions between neuroendocrine signaling and the circadian clock exert effects across multiple levels of biological organizations. At the molecular scale, clock gene products, hormone biosynthetic enzymes, and neurotransmitter synthesis components function within dynamic feedback networks. At the cellular level, specialized clock neurons and endocrine cells integrate incoming sensory information to regulate the downstream motor and secretory pathways.

At the organismal level, these integrated mechanisms shape the timing of core behaviors including foraging, reproduction, dispersal, and defense. In honey bees, JH and octopamine interact to regulate the transition of workers from nurse bees to foragers: JH acts in part by increasing octopamine levels in the brain, and combined treatment with octopamine and a JH analogue produces a higher proportion of foragers than treatment with either compound alone.^113^ At the social level, socially mediated synchronization of circadian oscillators is essential for efficient colony function and individual fitness in social insects.^40^ For example, bumblebee colonies align their foraging rhythms with daily resource availability, integrating individual circadian patterns into a colony-level cycle that partitions tasks to optimize overall efficiency.^114^ Social factors further modulate these rhythms to synchronize the activities of all worker individuals.^115^ At the ecosystem level, these hierarchical interactions support the delivery of ecosystem services: the synchrony between pollinator activity and crop flowering boosts agricultural yield^116,117^ and the temporal alignment between predators/parasitoids and pest phenology improves the efficacy of biological control and lowers dependence on chemical inputs.^118,119^

Understanding these cascading relationships allows us to move beyond descriptive observation to predictive modeling of beneficial insect dynamics under variable agroecosystem conditions. This multilevel integrative relationship, stretching from the molecular clock all the way to ecosystem services, shows how perturbations at the genetic level are amplified step by step and ultimately influence pollination efficiency.

Alongside the hierarchical structure introduced above, an interconnected regulatory network governed by circadian clocks, endocrine hormones, and biogenic amines functions through multiple bidirectional pathways, which cannot be fully captured by a strictly hierarchical framework. These interconnections form the mechanistic basis through which environmental signals are transduced into coordinated behavioral and physiological responses. A schematic depiction of this integrated neuroendocrine–circadian network is presented in Figure 3. It highlights the reciprocal interactions among clock genes, hormonal pathways, and aminergic neuromodulators as well as how this network is entrained by external cues including light, temperature, and nutritional status.

**Figure 3:**
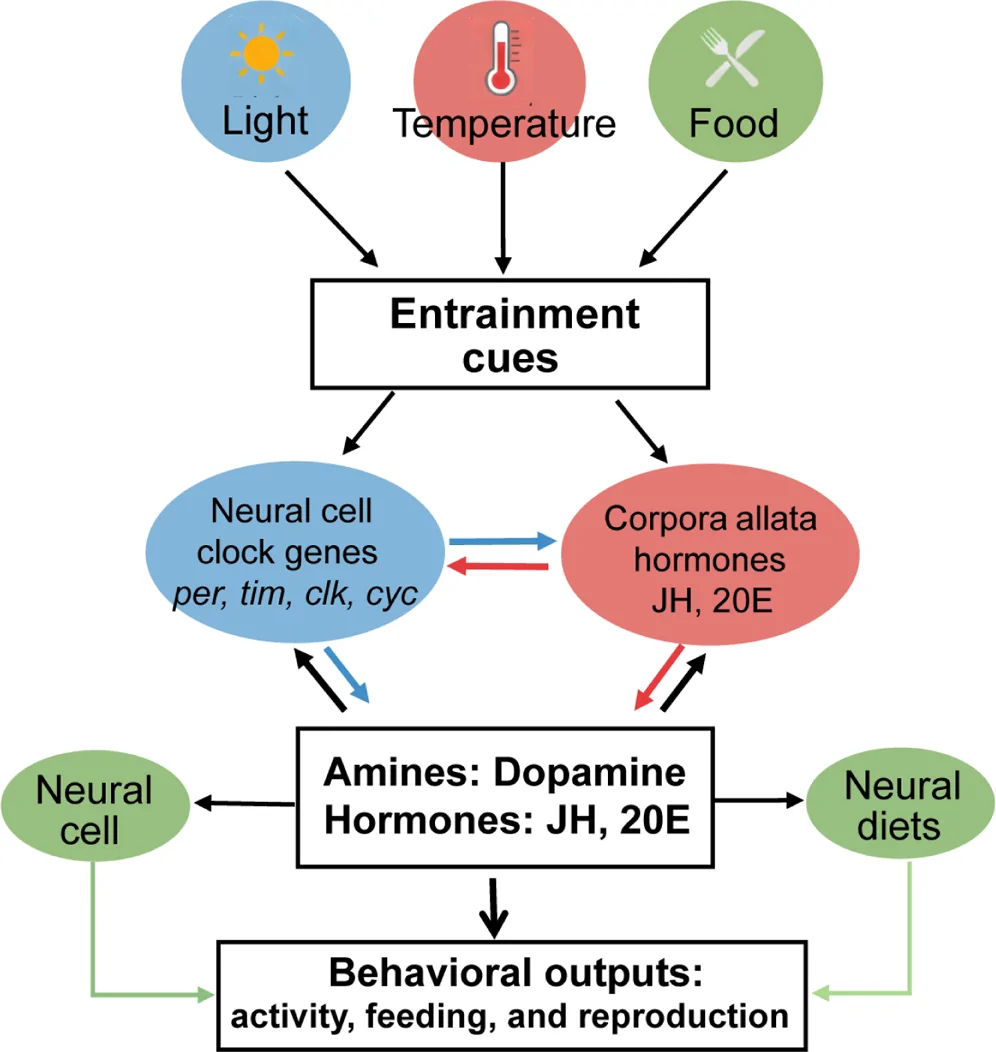
Schematic diagram of the neuroendocrine regulation of insect behavior. Environmental cues are processed via entrainment pathways that synchronize the expression of core clock genes and endocrine signals secreted by the corpora allata. Interactions between these genetic and hormonal components drive the production of biogenic amines and hormones, which ultimately regulate downstream behavioral outputs including activity, feeding, and reproduction.

## 4 Comparative Insights from Beneficial Insects

Having established the core mechanistic networks that underpin neuroendocrine–circadian integration, this section presents comparative case studies to illustrate how these regulatory systems are configured in two major functional groups of beneficial insects: natural enemies and pollinators (Figure 4).

**Figure 4:**
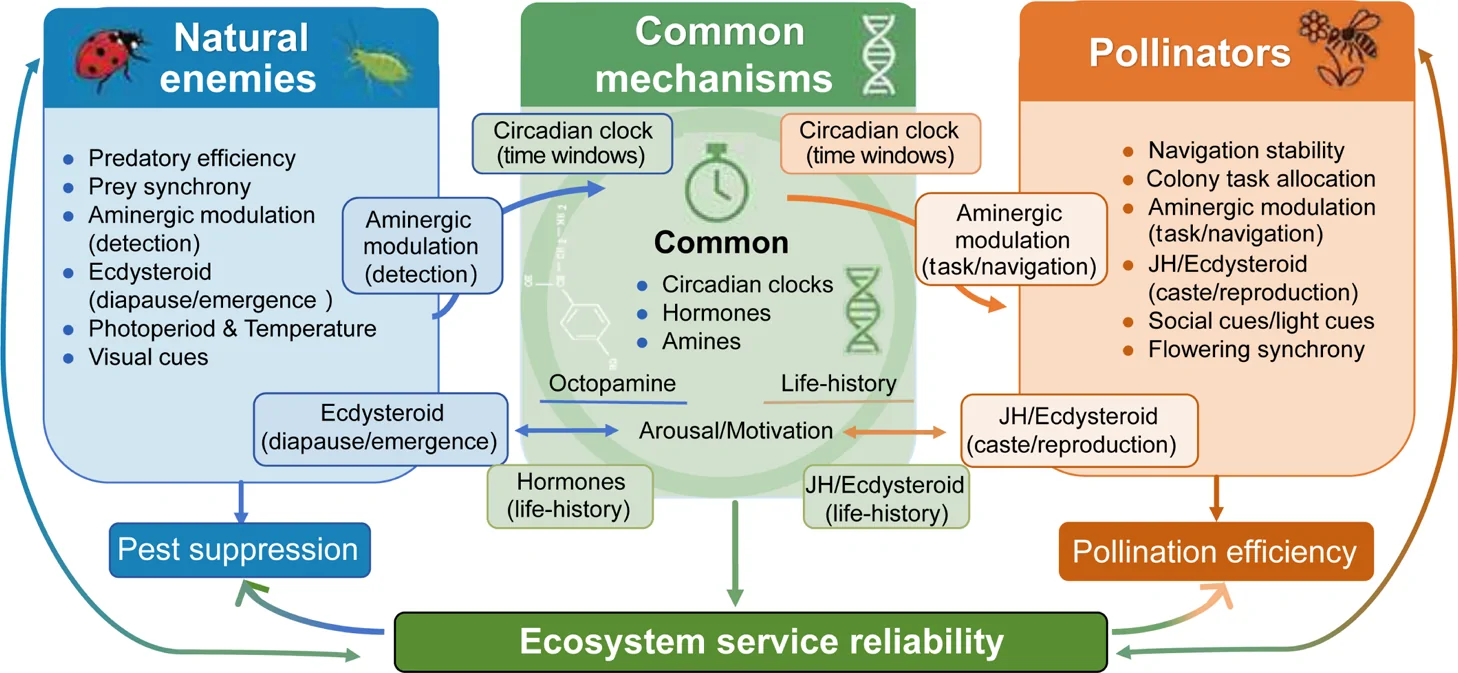
Diagram of the shared molecular and physiological mechanisms that underpin two key ecosystem services: natural pest control and pollination. Left Panel (Natural Enemies): Exocrine and endocrine pathways (amine modulation, ecdysteroid) regulate life-history traits including predatory efficiency, photoperiodic responses, and diapause, ultimately enhancing pest suppression. Right Panel (Pollinators): Circadian clocks and JH/Ecdysteroid signaling modulate navigation, colony task allocation, and reproduction to improve pollination efficiency. Center Panel (Common Mechanisms): The integration of circadian clocks (marked by a DNA icon) and hormonal signals (octopamine, JH, 20E) functions as a core regulatory switch for arousal, motivation, and life-history decision-making, guaranteeing the stable performance of these ecological functions.

### 4.1 Case Studies of Natural Enemy Insects

Natural enemies, including predatory beetles (Coccinellidae) and parasitoid wasps (*Trichogramma*), provide key biological control services by suppressing populations of herbivorous pests.^120–122^ Their behavioral ecology is tightly regulated via the integrated action of biogenic amines, endocrine hormones, and circadian clocks.

Octopamine and dopamine modulate predatory activity in lady beetles by regulating the locomotor behavior and responsiveness to prey detection. Experimental studies show that octopamine administration increases searching efficiency and promotes the entry of lady beetles into a predatory state, while dopamine modulates the transition between active and resting phases by altering the duration of rest bouts.^123,124^ These neuromodulatory effects are further constrained by circadian gating:^8^ peak predation activity usually aligns with the diel activity patterns of target prey such as aphids, which maximizes prey encounter rates (Figure 4).

### 4.2 Thermal Adaptation and Predator–Prey Synchrony under Climate Warming

Thermal adaptation is a critical determinant of natural enemy performance, influencing their efficacy in biological control programs. Studies on natural enemies such as *H. axyridis* and various parasitoid wasps show that rising temperatures can alter flight activity and predatory behavior.^125,126^ These behavioral shifts directly affect the synchrony between natural enemies and their prey or hosts. For example, temperature increases may enhance the narrow-range search capability in some predators while reducing their propensity for long-distance dispersal. Understanding species-specific thermal adaptations and the resulting synchrony or asynchrony with pest populations is therefore essential for predicting and improving the biological control efficacy under climate warming.

Synchronization between predator and prey populations, defined as the temporal alignment of predator activity with peaks in pest abundance, is largely mediated by the circadian clock. In *Trichogramma*, oviposition behavior is modulated by both circadian/photoperiodic cues and host egg age suitability. This ensures that parasitoids preferentially attack younger host eggs, when host vulnerability is the highest and the potential fitness of their offspring is the greatest.^78,127,128^ This predator–prey synchrony arises from the coordinated action of circadian clocks, endocrine signals (notably ecdysteroids), and amine-driven host-seeking behavior.

### 4.3 Case Studies of Pollinating Insects

Pollinating insects, particularly honey bees and bumblebees, possess tightly regulated circadian mechanisms that coordinate foraging activity with daily peaks in nectar and pollen availability, while task-related plasticity in these rhythms underpins the division of labor and sustains colony vitality.^22,129^

Synchronization with flowering phenology is primarily achieved through circadian entrainment, which allows pollinators to anticipate peaks in the availability of nectar and pollen. In honey bees, the circadian system interacts closely with endocrine signaling, and oscillations in clock gene expression are coordinated with hormonal pathways to regulate the temporal organization of behavior.^35^ This temporal organization is further refined by octopamine, which modulates sucrose responsiveness and foraging motivation.^10^ Even under unusual photoperiods such as the midnight sun, bumblebees still maintain robust diurnal foraging rhythms, with their circadian clocks entrained by daily cycles of light intensity and temperature.^77^

Pollinators maintain navigational precision via the circadian regulation of sun-compass orientation,^130^ as well as coordinated hormonal and aminergic networks that sustain flight endurance, with octopamine and JH being particularly critical.^131,132^ The Vg-JH module regulates energy allocation during the transition from nursing to foraging in honey bees,^133^ while octopamine signaling modulates foraging capacity by supporting flight muscle performance in insects.^134^ Disruption of circadian rhythms caused by exposure to artificial light at night can impair navigational accuracy and reduce pollen intake, demonstrating that coordinated regulation among circadian, endocrine, and neuromodulatory systems is required to achieve an optimal pollination.

In eusocial pollinators, the division of labor relies on the synergistic regulation of JH and dopamine. In honey bees, rising JH levels trigger the behavioral transition from nursing to foraging,^69^ while dopaminergic signaling modulates individual responsiveness to foraging-related cues.^135^ Bumblebees feature a highly plastic reproductive division of labor that is regulated by epigenetic mechanisms,^136^ reflecting species-specific integration of regulatory pathways: in honey bees, JH influences age-associated division of labor, while size-related division of labor in bumblebees is independent of JH, even though the two species share a common genetic toolkit^137^ (Figure 4).

### 4.4 Commonalities and Distinctions in Regulatory Mechanisms between Natural Enemies and Pollinators

Across both functional groups, circadian clocks delineate temporal windows for peak activity during prey capture or nectar foraging, while endocrine and neurochemical systems refine motivational and physiological readiness (Figure 4). Octopamine acts as a central modulator of arousal and motivation in both predators and pollinators. Dopamine supports learning, task flexibility, and motivational shifts, whereas serotonin synchronizes behavioral onset with prevailing light–dark cycles. Furthermore, individuals within a population exhibit consistent behavioral differences, known as behavioral syndromes, which represent another key dimension for comparison between the two groups.^138^ JHs and ecdysteroids regulate essential life history transitions, ranging from worker role differentiation in honey bees to diapause termination in lady beetles, and their activity is modulated by seasonal cues such as photoperiod and temperature. Oscillations of core clock genes coordinate daily rhythms of hormone secretion and neural excitability, keeping behavioral patterns synchronized with the environmental cycles.

Despite shared regulatory architectures, the modulation of these systems reflects distinct ecological imperatives. In natural enemies, octopaminergic signaling enhances the visual and olfactory sensitivity that is critical for prey detection, while ecdysteroid surges regulate diapause and adult emergence to align with the prey life cycles. Circadian gene expression is calibrated to match host or pest phenology, ensuring an accurate timing of hunting and oviposition. In contrast to natural enemies, neuroendocrine regulation in pollinators is strongly shaped by social organization and navigational requirements: JH times the nurse-to-forager transition and acts upstream of octopamine,^113,139^ while octopamine in the optic lobes modulates light responsiveness to meet the visual demands of foraging.^83^ Octopamine sustains foraging drive and navigational accuracy, dopamine supports colony-level division of labor, and JH fluctuations are elicited by pheromonal signaling and floral-resource dynamics. Seasonally, eusocial pollinators adjust colony demography via JH signaling: spring brood rearing is accelerated to match early floral blooms, whereas an autumn decline in JH triggers the physiological preparation of workers for overwintering. Circadian systems in pollinators are more closely aligned with floral phenology than with prey cycles,^42^ and navigational circuits rely extensively on polarized light, requiring circadian time compensation for accurate sun-compass orientation.^22,130^ These diel navigational rhythms are further calibrated by seasonal changes in solar elevation and day length, ensuring orientation accuracy across varying photic environments.

Integration of neuroendocrine and circadian mechanisms directly influences the reliability of the ecosystem services. In predators, disrupted synchrony with the prey phenology can reduce the efficacy of pest suppression. In pollinators, a temporal mismatch with floral resource availability leads to declines in pollination performance. Elucidating convergent and divergent regulatory traits thus provides a foundation for evidence-based conservation and management strategies. These insights can inform targeted interventions, including optimization of crop planting schedules or modification of artificial light regimes, to help sustain activity patterns aligned with the ecological demand for each functional group. These interactions are depicted in Figure 4, summarized by ecological category in Table 4 (Panel A: beneficial taxa; Panel B: target pest taxa), and detailed through mechanistic comparison in Table 5.

**Table 4:** Key Regulatory Traits and Ecological Adaptations across Functional Groups of Agroecosystem Insects

Functional group	Key regulators	Typical behaviors	Adapted environments	Primary ecological service	References
Panel A: Beneficial insects (providers of ecosystem services)					
Honey bee (*A. mellifera*)	JH	Foraging, navigation, and division of labor	Temperate and subtropical agroecosystems	Crop pollination	^70^
Bumblebee (*Bombus* spp.)	Photoperiod and temperature cues	Flexible foraging and behavioral plasticity	Temperate and alpine meadows	Pollination under variable climatic conditions	^77^
Lady beetle (*H. axyridis*)	Clock genes (*clk*/*cyc*), JH	Aphid predation, diapause regulation, and dispersal	Temperate farmlands	Biological pest control	^89^
Parasitoid wasp (*Trichogramma* spp.)	Clock genes (*clk, cyc, per, cry2, timeout*) regulated by photoperiod	Host location and synchronized oviposition	Diverse agroecosystems	Parasitic regulation of crop pests	^78,140^
Panel B: Target pest taxa (not ecosystem-service providers)					
Cotton leafworm (*Spodoptera litura*)	Photoperiod and temperature cues	Nocturnal feeding, mating, egg hatching	Tropical/subtropical agroecosystems	Target for pest suppression	^141^
Green peach aphid (*Myzus persicae*)	Photoperiod and temperature cues	Cyclical parthenogenesis	Temperate greenhouse/field systems	Target for pest suppression	^142^

**Table 5:** Cross-Species Distinctions in Neuroendocrine–Circadian Regulation between Pollinators and Natural Enemie**s**

Regulator or genes	Pollinators (e.g., *Apis*, *Bombus*)	Natural enemies (e.g., *Harmonia*, *Trichogramma*)	References
Octopamine	Enhances sucrose responsiveness; modulates reward valuation in dances	Modulates prey detection and locomotor pursuit (indirect)	^10,63^
Dopamine	Dopamine levels in the optic lobe undergo light/dark-cycle-dependent fluctuations	Modulates adaptive behavioral transitions (indirect)	^92^
Serotonin	Modulates circadian-entrained behavioral outputs	Modulates the circadian phase and aggression (indirect)	^35^
JH	Regulates the nurse-to-forager behavioral transition; seasonal JH titer changes align with colony activity	Coordinates reproductive diapause with prey abundance via the *clk/cyc*→JH axis	^70,89,143^
20E→ JH-mediated	Plays a secondary role in adult behavioral modulation	JH-mediated diapause regulation coordinates reproductive timing with prey abundance	^89^
*per*	*per* mRNA expression modulated by octopamine; aligns with daily activity rhythms	*per* expression photoperiodically regulated, synchronizing with host/pest timing	^28,78^
*tim*→ timeout	Bees possess timeout, not timeless; no *Drosophila* Tim1 ortholog	Wasps possess timeout; expression photoperiodically regulated	^78,88^
*clk*	Maintains colony circadian rhythm stability; CYC oscillates the antiphase to PER	*clk* expression photoperiodically regulated, controls adult emergence timing	^78,88^
*cry*	Possesses mammalian-type *cry-m*; *cry* mRNA oscillates with strong amplitude in foragers	Modulates light sensitivity underlying the predation cycle timing (indirect)	^35,88^

The divergence in neuroendocrine–circadian regulation between pollinators and natural enemies reflects evolutionary adaptations shaped by distinct ecological niches. For example, the prominent role of octopamine in driving pollinator foraging motivation aligns with eusocial selection for collective efficiency, whereas in solitary predators, dopamine-mediated dispersal prioritizes individual prey tracking. These differences are underpinned by life-history trade-offs: social insects such as honey bees exhibit JH-driven task partitioning optimized for stable colonies, while predatory beetles rely on ecdysteroid surges to enable rapid diapause termination in response to ephemeral prey outbreaks. This evolutionary perspective clarifies how conserved molecular toolkits are calibrated by natural selection to meet distinct ecological demands.

### 4.5 Evolutionary basis of Neuroendocrine–Clock Calibration: Life-History Strategies

The divergent neuroendocrine–circadian regulation between pollinators and natural enemies (Table 5) reflects life-history-driven selection acting on different regulatory time scales. We identify three axes through which the life-history strategy shapes the calibration of the clock–hormone–amine network.

#### 4.5.1 Solitary vs Eusocial: the Recruitment of JH as a Behavioral Pacemaker

In solitary insects, JH primarily functions as a gonadotropic signal that regulates the level of oogenesis. The evolution of eusociality in bees modified this linkage: in *A. mellifera*, JH no longer regulates adult oogenesis in worker bees but instead controls the age-related transition from in-nest brood care to external foraging. Topical application of the JH analogue methoprene accelerates this transition in a dose-dependent manner, establishing JH as the endocrine pacemaker of labor division.^85^ This aligns with the split-function hypothesis, which posits that JH originally regulated both reproduction and behavioral maturation in the ancestral solitary species. When reproductive and brood-care tasks became divided between queens and workers, the functions of JH became partitioned: it regulates reproductive maturation in queens and governs behavioral maturation (age polyethism) in workers.^144^ Consequently, the eusocial life history strategy, characterized by worker sterility, colony-level selection, and extended lifespan, favored the evolution of a slow, socially entrainable endocrine rhythm coordinated across thousands of individuals. The circadian clock provides the daily framework for this JH-driven ontogeny, in which *per* mRNA oscillates in phase with JH titers in foragers.^91^

#### 4.5.2 Parasitic vs Predatory: Neuropeptide-Mediated Instant Decision Gates

Solitary parasitoid wasps face a selective regime in which fitness depends entirely on locating host eggs within a narrow temporal window because freshly laid eggs remain vulnerable for only a few hours. Under such time-critical selection pressure, the neuroendocrine network must support rapid go/no-go decisions. Neuropeptides are ideal regulatory layers for this function. Genome-wide characterization of the endoparasitoid Pteromalus puparum identified 36 neuropeptide precursor genes, the lowest number among all examined insect species, which may be associated with its parasitic life cycle.^145^ Similarly, in the ectoparasitoid Habrobracon hebetor, 34 neuropeptide precursors and 44 receptors form a specialized signaling toolkit that differs from that of other parasitoids.^146^ These findings indicate that the neuropeptide toolkit in parasitoids is tightly coupled to their parasitic life history, providing the molecular basis for fast, context-dependent host seeking decisions. The circadian clock gates this neuropeptide-driven output to the photophase, the period when host eggs are freshly laid.^78^

In contrast, predatory beetles such as *H. axyridis* are solitary and polyphagous; their fitness depends on seasonal synchronization with aphid outbreaks. Accordingly, their neuroendocrine architecture retains JH and ecdysteroid as the primary regulators of diapause entry/termination and dispersal, with the CLK/CYC–NuA4/TIP60 complex converting photoperiod signals into JH-mediated diapause gating.^89^

#### 4.5.3 Specialist versus Generalist Calibration of Circadian Gating

The precision of circadian gating is correlated to the degree of dietary specialization. Monophagous parasitoids show sharper, photoperiod-entrained clock gene expression that synchronizes adult emergence and oviposition with host phenology.^78^ In contrast, polyphagous generalists such as lady beetles and bumblebees maintain broader circadian flexibility, enabling activity under variable thermal and photoperiod conditions.^77^ At the molecular level, this difference corresponds to differing reliance on two genes: timeout, which is present in parasitoids for precise photoperiod measurement, and cryptochrome, which is used in bees for light-entrained navigation.^35,88^ Thus, a specialist life history drives high-precision clock gating, whereas a generalist life history preserves circadian and endocrine plasticity.

### 4.6 Coupling between Pest Circadian Rhythms and Beneficial Insect Activity

The efficacy of ACM hinges on tripartite temporal coupling among pests, beneficial insects, and crop phenology as cross-trophic rhythm mismatches can undermine management outcomes. For natural enemy–pest systems, the synchronization of parasitoid adult emergence with host egg-laying peaks or of predator foraging peaks with aphid activity periods directly determines biocontrol success. For example, Trichogramma spp. restricts oviposition to a narrow early window after host egg deposition, and parasitism success declines sharply as host eggs age.^147^ This temporal restriction is under circadian control: *T. principium* females initiate and complete oviposition predominantly during the photophase, and these rhythms dampen under constant light.^140^ At the molecular level, *T. brassicae* possesses a functional clock gene network whose expression is photoperiodically regulated,^78^ confirming the mechanistic basis for how egg parasitoids synchronize their attacks with the vulnerable early embryonic stage of their hosts. Similarly, ladybird predation exhibits distinct diel rhythmicity, with peak consumption occurring during daytime hours.^86^ Photoperiod-induced reproductive diapause in *H. axyridis* is governed by JH signaling,^86^ and its termination is tightly coupled to aphid prey availability: reactivation requires consumption of protein-rich food and occurs at lower prey density thresholds in spring and summer when aphid abundance rises, reducing the risk of starvation.^87^

Climate warming increases the risk of desynchronization between natural enemies and prey. Unseasonably warm winters can trigger premature diapause termination in predatory insects. For example, gradually rising temperatures shorten the time to diapause termination in *Trichogramma dendrolimi*,^97^ while warm microclimates advance the seasonal dynamics of *H. axyridis*.^148^ In *H. axyridis*, diapause termination strictly depends on consumption of aphid prey,^87^ yet aphids are typically scarce in early spring, leading to postoverwintering starvation.^149^ This seasonal asynchrony is further compounded by diel mismatches: warm winters can advance the daily activity onset of predators, decoupling their foraging peaks from aphid diurnal movement patterns. Premature reactivation before pest populations rebound thus erodes the efficacy of biological control.^99^ Analogous seasonal mismatches occur in pollinators: earlier spring warming can advance colony foraging activity before local floral anthesis, reducing pollen availability and slowing colony growth. These cross-trophic seasonal disruptions indicate that diel clock alignment alone is insufficient for effective ACM, requiring the integration of long-term climatic rhythms into the management planning.

For pollinator–pest systems, temporal niche separation creates a low-intervention management window. Many lepidopteran pests (e.g., *Spodoptera litura*) are strictly nocturnal, with minimal activity overlap with diurnal pollinator foraging. Leveraging this rhythm-based separation allows targeted pest control (e.g., pheromone trapping, microbial insecticide application) during nighttime hours, when pollinators are inactive, to reduce nontarget mortality.^150^ Conversely, rhythm mismatches can give rise to unintended trade-offs: for example, artificial light at night disrupts both pollinator navigation and nocturnal pest activity, potentially increasing pest pressure while reducing pollination services. Optimizing ACM therefore requires explicit accounting of pest rhythms, alongside those of beneficial insects, to avoid counterproductive outcomes.

## 5 Application Frontiers

Before detailing specific intervention strategies, it is essential to define the operational criteria that classify a practice as ACM. For a management approach to be categorized as ACM, it must satisfy all of the following conditions:

C1. Temporal alignment: The intervention demonstrably shifts or synchronizes the diel or seasonal activity of a beneficial insect (or disrupts pest activity) relative to a given crop stage or pest-vulnerability window.

C2. Environmental cue manipulation: This strategy involves deliberately altering entraining cues, such as light, temperature, and nutrition, to modulate the insect clock and hormone systems.

C3. Multitrophic consideration: The phenology of pests, beneficial insects, and crops is evaluated jointly to avoid counterproductive outcomes.

C4. Evidence-based mechanisms: The intervention is justified by empirical data on circadian, hormonal, or aminergic regulation of the target species.

In this section and Table 6, we distinguish mechanistic compatibility from field readiness. Mechanistic compatibility denotes whether an intervention can affect circadian–hormonal–aminergic timing. Field readiness means whether it has been validated and risk assessed under real agroecosystem conditions. High mechanistic compatibility does not mean that there is high field readiness. The following subsections describe interventions that meet these criteria to varying degrees, as indicated in Figure 5 and Table 6.

**Table 6:** Intervention Strategies, Mechanistic Compatibility, Operational/Field Readiness, Benefits, and Risks^a^

Strategy	Potential benefits	Risks and regulatory challenges	Rhythm-based management compatibility	Operational/field readiness	References
Photoperiod and thermal control	Synchronizes activity cycles with crop phenology; relatively low-cost infrastructure in controlled environments	Risk of maladaptation if artificial cues diverge from natural environmental signals; limited scalability in open-field systems	High in controlled environments; moderate in open-field systems. Can be calibrated to align beneficial insect activity with pest susceptibility peaks while minimizing overlap with nontarget species	Moderate in controlled environments; low–moderate in open-field systems. Requires adaptive scheduling, environmental monitoring, and seasonal climate forecasting	^97,99,151,153^
Nutritional modulation	Enhances target behaviors through dietary supplementation; can increase foraging motivation, learning ability, or predatory motivation	Possible nontarget impacts on other species; requires precise nutrient formulation; efficacy depends on delivery context; regulatory frameworks for neuroactive supplements remain unclear	Moderate in controlled/semifield settings; low–moderate in open-field systems. Requires formulation to avoid stimulating pest activity	Moderate in controlled/semifield settings; low in open-field systems. Long-term stability and field validation remain limited	^10,63,163,170^
Molecular targeting and gene editing	Enables precise modification of clock genes and behavioral phenotypes; potential simultaneous enhancement of beneficial insect clock function and disruption of pest clock systems	Stringent regulatory oversight; substantial ecological risk; variable public acceptance; scarce field validation; concerns about gene flow and trait stability across generations	High mechanistic compatibility. Strong mechanistic evidence, but context-dependent and not yet field-validated at scale	Low–moderate. Regulatory, ecological, and public-acceptance barriers remain major constraints	^157,164,171^
Synthetic ecology and multispecies complementary networks	Leverages temporal niche partitioning across pests, beneficial insects, and crops; can integrate pollinators and natural enemies to sustain services throughout the growing season	High management intensity; potential interspecific competition; scaling challenges; requires rigorous monitoring and adaptive oversight	High conceptual compatibility. Explicitly uses temporal niche partitioning, but outcomes depend on local species assemblages and management capacity	Moderate. Promising in greenhouses and diversified systems, but scaling to heterogeneous landscapes remains difficult	^166,172–174^

a Definition of the grades used in the Rhythm-based management compatibility and Operational/field readiness columns. Each grade integrates four criteria: (^1^ mechanistic evidence linking the intervention to circadian, hormonal, or aminergic regulation; (^2^ demonstrated ability to alter the diel or seasonal activity of the target insects; (^3^ scalability in the relevant production system; and (^4^ ecological and regulatory risks. Very High: field-validated in open agroecosystems with low or monitorable risk. High: validated in controlled or semifield systems and/or supported by strong mechanistic evidence, with manageable risk. Moderate: supported by laboratory or small-scale evidence, context-dependent, with unresolved scalability or risk-control issues. Low: conceptual or theoretical, with limited field evidence and high regulatory and ecological uncertainty. Because feasibility differs between open-field and controlled environments, grades are reported separately for each where necessary; a “High” under controlled conditions does not equate to a “High” in open-field environments.

**Figure 5:**
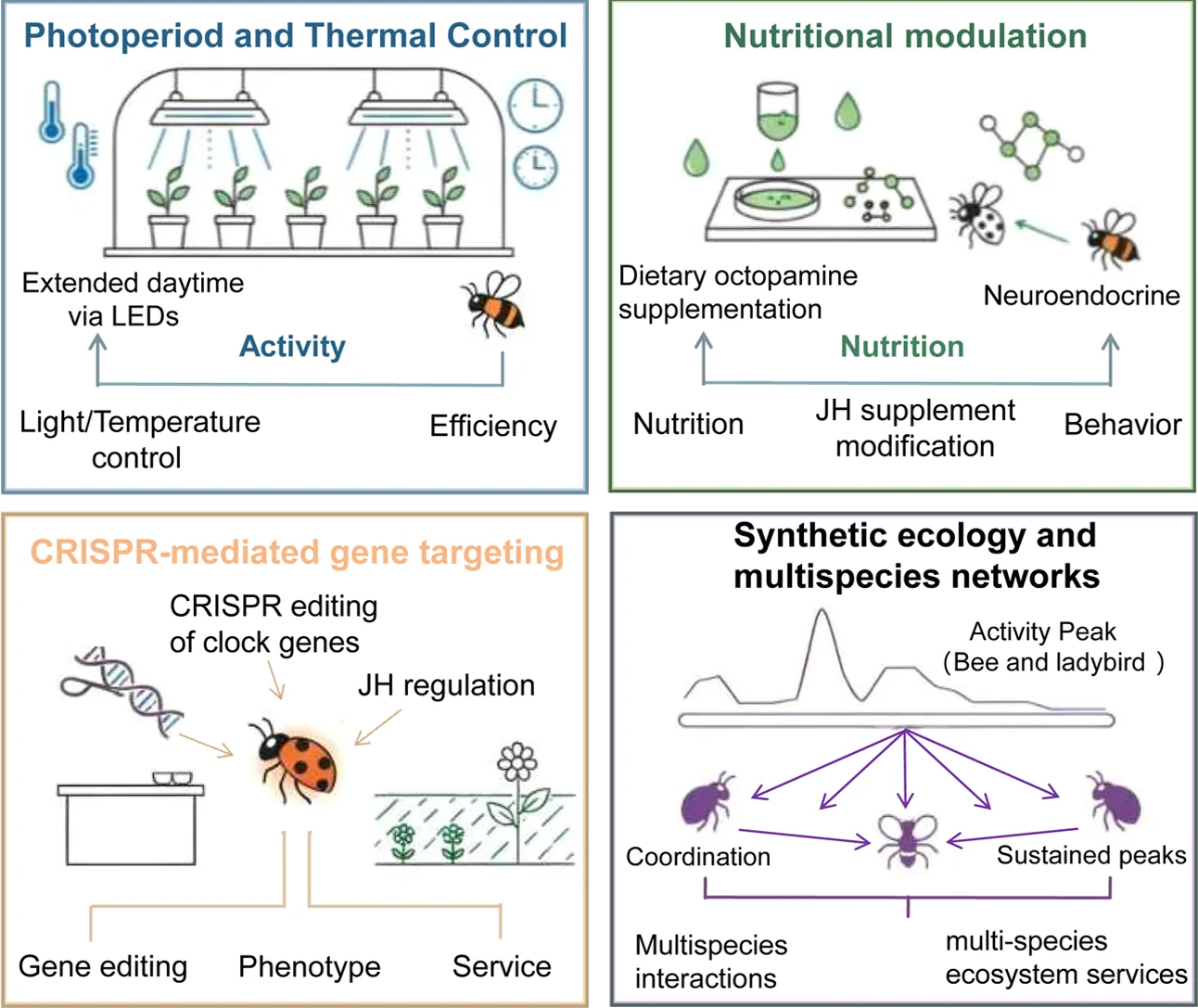
Application scenarios of the agricultural chrono-management strategy. This schematic outlines four core intervention approaches: (^1^ light and temperature manipulation to synchronize the activity of beneficial insects with crop phenology; (^2^ nutritional and neuroendocrine regulation through dietary components or exogenous JH to improve behavioral efficiency; (^3^ molecular targeting via CRISPR technology to edit clock genes, enabling precise modification of insect phenotypes and ecosystem services; and (^4^ synthetic ecology approaches that utilize temporal niche partitioning in multispecies communities (e.g., diurnal bees and lady beetles versus nocturnal pests) to optimize the delivery of ecosystem services.

### 5.1 Photoperiod and Thermal Intervention for Flowering or Pest Emergence

Manipulating photoperiod and temperature is an effective strategy for synchronizing the activity patterns of beneficial insects with agricultural requirements. Circadian clocks in pollinators and natural enemies are highly plastic under artificial light (Figure 3), aligning foraging and predatory peaks with crop flowering and pest emergence. In honey bees, clock reorganization and illuminance responses synchronize foraging with floral rhythms.^22,151–154^ In natural enemies, photoperiod and scotophase light modulate pest behavior and life cycles, cascading to predator–prey synchrony.^24^ In greenhouse pollination using *B. terrestris*, artificial light regimes shape foraging activity in a nonlinear manner: foragers retain an endogenous circadian window;^77^ deviations in light intensity or spectrum reduce foraging activity and success in homing,^155^ while supplementation with ultraviolet light helps maintain foraging responsiveness. While manipulating photoperiod and temperature is a promising approach to align beneficial insect activity with agricultural needs, its implementation at field scale faces significant constraints. The primary limitation is the low scalability of precise environmental control in open-field systems, where climatic variability and habitat heterogeneity can disrupt targeted neuroendocrine–circadian processes. In controlled environments such as greenhouses, extending daylight hours through low-intensity supplementary lighting has already proven effective. For example, in *B. terrestris* pollination systems, prolonged daylight enhances colony-level foraging activity and overall pollination efficiency.^77,156,157^ Similarly, controlled prerelease temperature treatments can synchronize the emergence of mass-reared predators with local pest population peaks by mimicking seasonal diapause termination cues. Nevertheless, excessive or poorly timed manipulation may trigger temporal mismatches under variable climates, requiring adaptive scheduling, continuous environmental monitoring, and integration of seasonal climate forecasting to avoid premature reactivation of beneficial insects before prey populations are established. As evaluated in Table 6, photoperiod and thermal interventions have high rhythm-based management compatibility in controlled environments but only moderate compatibility in open-field systems. They can synchronize beneficial insect activity with pest susceptibility peaks but carry risks of maladaptation when artificial cues diverge from natural environmental signals and remain difficult to scale in open-field systems. For example, photothermal manipulations can synchronize *Trichogramma* activity with pest susceptibility windows while minimizing nontarget effects on managed pollinators. Adult emergence of this parasitoid is photoperiodically plastic: it peaks shortly after light onset and does not occur during the scotophase.^158–160^ Accordingly, brief supplemental lighting in the evening shifts this parasitoid’s activity to align with the nocturnal oviposition window of lepidopteran pests such as *Pectinophora gossypiella*,^161^ without disrupting the diurnal foraging of greenhouse bumblebees, which is limited to daytime hours.^162^

### 5.2 Nutritional and Neurochemical Feeding Strategies

Targeted nutritional interventions offer a minimally invasive pathway to enhance the behavioral performance of beneficial insects via neurochemical modulation; however, their practical applications face notable constraints. Supplementation with carbohydrate-rich diets or compounds that influence octopaminergic/dopaminergic signaling can increase foraging motivation and learning ability in honey bees,^10^ while protein enrichment can raise JH titers to accelerate the nurse-to-forager transition.^163^ Prey mimics or nectar sources enriched with neuroactive compounds, such as plant-derived alkaloids, can similarly prime predatory or parasitoid behaviors without requiring genetic manipulation. However, potential nontarget consequences must be carefully evaluated: dispersal of treated insects beyond target areas could alter local food web interactions and ecosystem dynamics, and nutrient formulations must be designed to avoid inadvertently stimulating pest activity. Furthermore, the long-term stability of diet-induced behavioral changes and the absence of clear regulatory frameworks for neuroactive supplements in open agroecosystems create additional hurdles. Pairing neurochemical supplements with pest susceptibility peaks (e.g., periods of low detoxification enzyme activity) can help mitigate these risks, but rigorous field validation remains essential. As summarized in Table 6, neurochemical feeding strategies have moderate rhythm-based management compatibility in controlled or semifield settings and low-to-moderate compatibility in open-field systems. They can enhance target behaviors such as foraging favor or predatory motivation but require precise nutrient formulation and controlled delivery to avoid nontarget impacts on other species.

### 5.3 Molecular Targeting and Gene Editing

Although CRISPR-Cas9 and other molecular tools have transformative potential for optimizing beneficial insect performance, significant technical, regulatory, and socioecological constraints limit their near-term applications. Recent research has demonstrated that disrupting or modifying core clock genes can shift the circadian phase and amplitude in model insects,^25^ and targeted receptor modification could theoretically increase responsiveness to floral volatiles or pest pheromones. However, field implementation carries substantial risks: gene flow from released insects to wild populations could spread edited traits into nontarget species and reshape community dynamics, behavioral modifications may diffuse unpredictably across heterogeneous landscapes, and the stability of engineered traits across successive generations under fluctuating thermal and photoperiodic conditions remains unproven. From a regulatory perspective, gene-edited insects destined for environmental release are subject to stringent oversight, including Directive 2001/18/EC in the European Union and APHIS evaluation in the United States, which require comprehensive ecological risk assessment. Public perception also represents a barrier: surveys show greater public acceptance of medical or conservation applications of gene editing than of agricultural uses directly linked to food production.^164,165^ Establishing contained semifield experiments, rigorous postrelease monitoring, and adaptive management protocols is therefore a prerequisite for any open-environment deployment. As detailed in Table 6, molecular targeting has high mechanistic compatibility with rhythm-based management, enabling simultaneous enhancement of beneficial insect clock function and disruption of pest clock systems. However, its current operational/field readiness is low to moderate because of stringent regulatory oversight, variable public acceptance, substantial ecological risk, and scarce field validation.

Beyond the ecological and regulatory risks outlined above, ensuring regulatory compliance, transparent risk communication, and stakeholder participation will be critical to balancing technological innovation with ecological integrity and sustaining public confidence. Complementary strategies that target pest clock genes (e.g., CRISPR editing of *per* or *tim* to disrupt diapause termination or mating rhythms) are an emerging approach to strengthen ACM, although these methods face the same ecological and regulatory risks as edits introduced to beneficial insects.

### 5.4 Synthetic Ecology and Multispecies Complementary Networks

Synthetic ecology frameworks aim to construct agroecosystems where multiple beneficial species function in a coordinated, mutually reinforcing manner, but their implementation is constrained by high management complexity and potential ecological risks. Integrating clock-entrained pollinators with predators or parasitoids that target pests at different developmental stages can sustain ecosystem services throughout the entire growing season. For example, deploying early active bumblebees alongside midday-foraging honey bees expands pollination coverage, while synchronizing parasitoid oviposition with predator hunting peaks reduces the likelihood of pest escape.^166,167^ Realizing these systems requires coordinated management of photoperiod, temperature, and nutritional resources to entrain each species’ neuroendocrine–circadian architecture and achieve precise temporal niche partitioning. However, unbalanced population dynamics or resource competition may arise and can potentially lead to trophic imbalances if not carefully monitored. Adaptive management protocols and continuous performance assessment are therefore essential. Practical demonstrations, such as those conducted in Japanese tomato greenhouses (*B. terrestris* with *H. axyridis*) and Mediterranean citrus orchards (*A. mellifera* with *Trichogramma cacoeciae*), have shown promising results, but scaling these networks to heterogeneous landscapes remains a major challenge. As detailed in Table 6, synthetic ecology has high conceptual compatibility with rhythm-based management because it explicitly leverages temporal niche partitioning across pests, beneficial insects, and crops. However, its current operational feasibility is moderate, given high management intensity, potential interspecific competition, and scaling challenges; rigorous monitoring and adaptive oversight are required.

Illustrative applications, including optimized greenhouse pollination, targeted biological control releases, and multispecies service networks, are presented in Figure 5. Temporal niche partitioning is a core component of synthetic agroecology. Managed bumblebees and aphidophagous coccinellids forage during the day,^162,168^ avoiding overlap with nocturnally ovipositing pests such as *P. gossypiella*.^161^ This diurnal–nocturnal partitioning supports simultaneous pollination and pest suppression, an effect that is further strengthened by floral diversification to boost natural enemy populations.^169^

### 5.5 Climate Change as a Pervasive Constraint on Application Efficacy

Beyond the strategy-specific constraints identified above, climate change acts as a pervasive, cross-cutting constraint that further limits the efficacy of all ACM interventions. Gradual and acute climatic perturbations can disrupt coordination between the neuroendocrine and circadian systems. Elevated temperatures accelerate metabolic processes and induce circadian phase advances, shifting peak activity earlier in the day or season. Although such adjustments may transiently improve synchrony with early resource availability, they also increase the risk of desynchronization from historically reliable environmental cues, such as floral anthesis for pollinators or prey emergence for natural enemies. Sustained warming trends may therefore erode the adaptive stability of established clock–hormone–amine regulatory frameworks.

Seasonally regulated endocrine cycles, particularly those involving JH and ecdysteroids, are also vulnerable to a temporal mismatch with resource availability. Mild winters disrupt the synchrony between predators and their prey, which undermines the efficacy of the biological control. Rising temperatures cause a significant increase in aphid density yet reduce the predatory efficiency of lady beetles;^175^ these temperature increases also deplete the metabolic reserves of coccinellids.^176^ In pollinators, altered hormonal rhythms may desynchronize brood development from nectar and pollen availability peaks, reducing the colony productivity and survival. These seasonal mismatches are exacerbated by extreme climatic events: heatwaves during late winter can prematurely elevate JH titers in both predators and pollinators, while unseasonal frosts can delay floral anthesis or kill early season prey, creating persistent trophic asynchrony that lasts from weeks to months. Such mismatches can propagate through trophic networks, undermining the ecosystem stability and the reliability of insect-mediated ecosystem services. Critically, the magnitude of these seasonal disruptions often outweighs that of diel rhythm misalignment in driving the ecosystem service loss. For instance, a substantial delay in floral blooming relative to pollinator foraging has a far greater impact on crop yields than a minor shift in the onset of daily foraging.

Extreme climatic events, including heat waves, heavy precipitation, and unseasonal frosts, can override circadian control mechanisms. Sudden thermal surges may disrupt biogenic amine homeostasis, reducing locomotor capacity and foraging efficiency, while exposure to temperature extremes can destabilize clock gene oscillations, impairing time-compensated navigation in pollinators and prey tracking in predators.^177^ These stress-induced perturbations can shift populations from stable service provisions to erratic population dynamics.

Mitigating climate-driven disruption is, therefore, a prerequisite for the effective implementation of neuroendocrine and circadian management strategies. Integrating climate responsiveness into intervention design, including photoperiod manipulation and synthetic ecological frameworks, is essential for developing resilient ACM systems that can sustain their function under environmental change. Climate change undermines the reliability of environmental cues; for instance, warmer winters can advance diapause termination before prey become available. Consequently, management strategies must actively entrain insect activity to buffer against such perturbations. In this context, multispecies complementary networks with staggered activity patterns can promote service continuity under climatic volatility consistent with ecological insurance principles.

### 5.6 Translational Barriers and Integrated Solutions

In addition to the ecological and technical constraints detailed in Sections 5.5, several translational barriers further limit the transition from controlled experimental frameworks to large-scale agricultural implementation. The following subsections provide a systematic assessment of these translational barriers alongside prospective strategies for their mitigation and integration into practical management frameworks.

#### 5.6.1 Technical and Ecological Barriers

The operational deployment of strategies derived from the ACM framework faces significant technical and ecological hurdles. A primary limitation to scalability is that many interventions, including precise photoperiods and thermal modulation, have mostly been validated under controlled laboratory conditions. Their efficacy often diminishes in open-field conditions due to climatic variability, habitat heterogeneity, and uneven resource distribution, all of which can disrupt targeted neuroendocrine–circadian processes. Furthermore, the persistence of engineered or environmentally induced traits across multiple generations in dynamic environments remains unverified, raising concerns about the long-term durability of these ecosystem services.

Ecological risks also require a careful consideration. Altered insect populations may interact with their wild counterparts and other species in unforeseen ways, potentially disrupting local adaptations and community structures. For example, gene flow from engineered strains can compromise the genetic integrity of native populations through the introgression of released-strain genomes.^178,179^ Meanwhile, behaviorally enhanced biological control agents may spread beyond their optimal foraging ranges via kairomone-mediated recruitment,^180^ potentially attracting nontarget organisms and altering prey dynamics as well as the ecological balance.

#### 5.6.2 Policy and Socioeconomic Barriers

Widespread implementation is further constrained by the policy and socioeconomic challenges. High capital requirements for specialized breeding facilities, controlled-environment infrastructure, and multispecies surveillance programs make such initiatives economically unfeasible for many agricultural enterprises, particularly small- to medium-scale farms. Successful application often requires coordinated participation among neighboring landholders to manage ecological spillover and preserve contiguous habitats, a goal that is difficult to achieve in fragmented landscapes. In addition, irregular funding streams and limited market incentives for ecosystem-service-based innovations discourage long-term investment.

From a policy perspective, regulatory authorization for gene-edited or -modified beneficial insects varies markedly across jurisdictions, reflecting divergent safety standards and levels of public acceptance. These processes are shaped not only by empirical risk assessment but also by public attitudes, cultural norms, and perceptions of the transparency and institutional credibility.

#### 5.6.3 Mitigation Pathways and Integrated Solutions

Overcoming these multifaceted constraints requires an integrated cross-sectoral approach. Translation from experimental insight into applied practice can be advanced through adaptive scaling: this approach uses phased field trials that progressively increase in complexity, paired with rigorous monitoring to identify and mitigate unforeseen ecological outcomes. In parallel, an economic innovation is essential. This includes developing cost-sharing mechanisms, ecosystem service markets, and public–private partnerships to reduce financial burdens and incentivize farmer participation. These efforts must be complemented by policy harmonization to align the international risk assessment protocols and regulatory frameworks.

Sustained stakeholder engagement and capacity building are also necessary. This involves engaging farmers, policymakers, and the public through participatory research, transparent communication, and targeted training programs. Adaptive management frameworks that integrate decision-making, monitoring, and iterative learning^181^ are critical for translating the promising concept of adaptive biological control management into reliable, field-ready applications. The large-scale propagation and release of Trichogramma in China exemplifies the multifaceted challenges of this scaling-up process,^122^ among which diapause regulation stands as a key biological dimension.^38^

## 6 Discussion

### 6.1 Research Gaps

Despite substantial progress in elucidating the neuroendocrine and circadian regulation of beneficial insects, major gaps remain when it comes to translating mechanistic insights into practical field-based applications. Existing knowledge on neurotransmitter function, hormonal modulation, and clock gene dynamics remains fragmented,^182,183^ which hinders research integration. We already know that JH biosynthesis involves neurotransmitters and neuropeptides acting through GPCRs^182^ and that circadian pacemakers transmit signals via pigment-dispersing factor (PDF).^183^ However, the cross-talk between these regulatory pathways is still poorly understood.^184^ Most investigations have centered on single-factor manipulations conducted under controlled laboratory conditions, leaving the interactive and synergistic effects of multiple environmental variables largely unexplored. A prominent knowledge gap exists in multiscale integration: specifically, we lack an understanding of how molecular perturbations propagate through physiological systems to shape population stability and the consistent delivery of ecosystem services. Although interactions between the circadian clock and JH have already been confirmed, as exemplified by the CLK–CYC–Met gut crosstalk reported in previous studies^185^ and period-dependent JH biosynthesis,^186^ how these interactions are regulated under natural field conditions remains poorly understood. A key limitation is that most existing studies of clock–hormone interactions focus exclusively on diel time scales, while the mechanisms through which seasonal photoperiodic and thermal cues recalibrate these daily interactions are almost entirely uncharacterized. For example, we do not yet know how long-term cold exposure during diapause alters the diel oscillations of JH biosynthetic enzymes or how seasonal changes in daylength modify clock gene expression in wild populations of beneficial insects. This limitation can be addressed through integrated multiomics approaches, including genomics, transcriptomics, proteomics, and metabolomics, implemented within a systems biology framework and paired with long-term longitudinal field sampling across multiple seasons and years to capture the full temporal spectrum of regulatory dynamics.

Clear delineation between species-specific and conserved regulatory pathways remains inadequate. Findings from model taxa such as Drosophila are frequently extrapolated to beneficial species, including honey bees and lady beetles, without adequate empirical validation. Adaptive traits linked to microclimatic tolerance or foraging specialization are rarely incorporated into comparative frameworks that account for real ecological diversity. Another critical limitation is the shortage of longitudinal field data sets. Short-term studies can capture immediate behavioral responses,^77^ but they often overlook interannual variability and cumulative climate impacts. Without sustained multisession observational data, predictive models of ecosystem service performance remain largely speculative. The provenance audit in Section 3 identifies this gap: among the 11 taxa included in the mechanistic network, only four (*A. mellifera*, *B. terrestris*, *H. axyridis,* and *Trichogramma* spp.) are themselves beneficial insects, and the links corresponding to the other seven remain to be directly verified in pollinators and natural enemies.

An additional gap lies in cross-functional research that covers both pollinators and natural enemies. Despite the equally important roles that both groups play in agroecosystem management, they are typically studied separately. The lack of an integrated conceptual framework that can optimize their combined ecosystem services under shared environmental constraints continues to hinder the development of holistic agricultural management strategies.

Research on neuroendocrine and circadian integration can offer insights that extend far beyond those of entomology. The bidirectional regulation between circadian clocks and hormone secretion in insects parallels the mechanisms of circadian disruption in human metabolic disorders, while biogenic amine regulation of foraging motivation mirrors dopamine-driven reward pathways in vertebrates. These parallels create opportunities for cross-phylum inference to advance our understanding of how climate-induced desynchronization impacts the animal fitness. Adapting this research framework to study migratory birds or marine species may help advance the development of a unified theory of behavioral resilience under environmental changes.

Finally, multitrophic rhythm coupling remains understudied across both diel and seasonal time scales. The rhythmic correlations among different trophic levels and their underlying mechanisms are still largely unknown,^24^ and existing multitrophic interaction frameworks in crop protection have predominantly focused on chemically mediated mechanisms rather than circadian coupling.^187^ To avoid unintended outcomes, such as inadvertently synchronizing pest outbreaks while seeking to enhance beneficial insect activity, predictive models of ecosystem service performance must incorporate multitemporal rhythmic interactions that span from hourly foraging decisions to seasonal diapause termination.

### 6.2 Evolutionary Implications and Phylogenetic Conservation

The regulatory networks outlined in this work are dynamic products of adaptive evolution. The conservation of clock genes such as *per* and *tim* across insects points to their ancestral functions in synchronizing behavior with cyclical environments, whereas lineage-specific modifications, such as *cry* expression that supports navigation in pollinators, reflect niche specialization. Future studies should adopt phylogenomic approaches to trace the coevolution of hormone receptors and clock genes and test hypotheses about how sociality or dietary shifts drive regulatory innovation. This line of research may clarify why some species, such as generalist bumblebees, retain behavioral plasticity under climate change, while specialists face elevated extinction risks.

### 6.3 Future Research Directions

Recent advances in genomics, transcriptomics, proteomics, and metabolomics have created unprecedented opportunities to untangle the complexity of regulatory networks in insects. Integrating these multiomics data sets within a systems biology framework will enable comprehensive mapping of the neuroendocrine–circadian–behavioral axis, linking genetic variation to physiological processes and ecological outcomes. For beneficial insects, this integration can help identify conserved molecular biomarkers associated with activity phase, stress tolerance, and reproductive state, supporting the development of standardized monitoring tools applicable across taxa.

The simultaneous development of machine learning and computer vision has further expanded research capacity, enabling continuous, noninvasive surveillance of insect behavior under field conditions.^188,189^ A particularly promising research direction is the convergence of artificial intelligence (AI), ecological engineering, and precision agriculture, as emphasized in recent studies on sustainable agroecosystem design. Combining environmental sensor data with AI-powered behavioral recognition systems can produce real-time forecasts of foraging and predatory activity. AIoT-based monitoring systems have demonstrated the feasibility of this integration by synchronizing deep-learning-based pest recognition with environmental sensing for continuous, real-time field deployment.^190^ Predictive algorithms trained on species-specific data sets can then dynamically adjust greenhouse lighting, natural enemy release timing, and resource provisioning to stabilize the delivery of ecosystem services under variable climatic conditions. AI-based analytics also holds promise for the early detection of physiological stress or functional decline at the colony and population scales, enabling rapid, preemptive management interventions.

Precision agriculture technologies provide practical tools for modulating environmental parameters on fine spatial and temporal scales, bridging the gap between laboratory discoveries and field implementation. Light-emitting diode (LED) lighting arrays, localized thermal control systems, and microirrigation networks can be used to tailor microclimatic conditions in targeted plots.^191^ For beneficial insects, this makes it possible to create spatially defined zones optimized for specific functional groups; for example, one could synchronize early floral anthesis with the activity of early foraging pollinators or align midday pest outbreaks with peak predator activity. High-resolution satellite and drone imagery can further coordinate these zones across landscapes, aligning insect behavior with crop phenology and pest development.

Future management approaches should integrate beneficial insect conservation and regulation into broader ecological engineering frameworks. This includes designing multispecies assemblages that can support pollination, pest suppression, and habitat restoration simultaneously. At the landscape scale, strategically incorporating wildflower strips, diversified crop mosaics, and seminatural refugia can improve resource continuity, stabilize population dynamics, and buffer environmental variability. Such integrated ecological networks are essential for developing agricultural systems resilient to both biotic perturbations such as pest outbreaks and abiotic stressors such as heat waves or drought, ensuring the sustained provision of ecosystem services under changing climatic regimes.^173^

### 6.4 Theoretical and Practical Significance

Integrating neuroendocrine and circadian biology into the study of beneficial insect ecology reshapes the conceptual foundation for ecosystem service provision. Ecosystem services such as pollination and predation are no longer viewed as static traits but rather as dynamic outputs from environmentally responsive regulatory networks. This synthesis establishes a multiscale paradigm for agricultural ecosystem design that connects molecular mechanisms to landscape-level processes. The resulting systems-oriented framework supports the development of predictive models capable of forecasting service performance under various environmental and management contexts.

These insights lay the scientific foundation for next-generation agricultural management. By targeting key regulatory nodes, including circadian phase alignment, hormone titers, and neurotransmitter cycling, it becomes feasible to synchronize insect activity with crop phenology and agronomic schedules. This level of precision has the potential to reduce dependence on chemical pesticides, boost crop productivity, and stabilize ecosystem service delivery during environmental fluctuations.

The integration of AI-enabled monitoring and precision intervention further enables real-time optimization of insect performance without requiring invasive manipulation.^192,193^ Embedding these technologies within ecological engineering frameworks extends their impact beyond single-crop systems, supporting biodiversity conservation while sustaining agricultural productivity.

At the global scale, adopting integrated neuroendocrine–circadian management frameworks may strengthen the resilience of agricultural systems to climate change, provided that these approaches are validated under field conditions and that open-field scalability and ecological risks are rigorously assessed. Beneficial insects regulated through these approaches may exhibit enhanced adaptive capacity, supporting the continuous provision of pollination, pest suppression, and related ecosystem functions under shifting temperature and photoperiod regimes. This contributes to diversified agroecosystems that are less vulnerable to a systemic disruption.

Ultimately, this review frames ACM as a core component of climate-smart agriculture. By harnessing neuroendocrine and circadian plasticity, we can design agricultural systems that anticipate environmental change and enhance ecosystem resilience rather than focus solely on output optimization. Extending the principles of internally timed adaptation from individual insects to the whole landscape provides a pathway to developing agricultural systems that buffer climatic uncertainty and advance global sustainability goals.
